# Genomic imprinting in an early‐diverging angiosperm reveals an ancient mechanism for seed initiation in flowering plants

**DOI:** 10.1111/nph.70776

**Published:** 2025-11-28

**Authors:** Ana M. Florez‐Rueda, Mathias Scharmann, Leonardo P. de Souza, Alisdair R. Fernie, Julien B. Bachelier, Duarte D. Figueiredo

**Affiliations:** ^1^ Max Planck Institute of Molecular Plant Physiology Am Muehlenberg 1 14476 Potsdam Germany; ^2^ Institute of Biochemistry and Biology University of Potsdam Karl‐Liebknecht‐Str. 24‐25 14476 Potsdam Germany; ^3^ Division of Biochemistry, Department of Biology Friedrich‐Alexander‐University Erlangen‐Nuremberg 91058 Erlangen Germany; ^4^ Institute of Biology Freie Universität Berlin 14195 Berlin Germany

**Keywords:** auxin, early diverging angiosperm, endosperm, genomic imprinting, gymnosperm, seed development

## Disclaimer

The New Phytologist Foundation remains neutral with regard to jurisdictional claims in maps and in any institutional affiliations.

## Introduction

The evolution of the seed habit marks a pivotal innovation of the spermatophytes. Angiosperms further refined this trait by coupling the development of seed accessory structures to fertilization, optimizing resource allocation. Here, we show that post‐fertilization auxin production is an evolutionarily conserved paternal trigger for the development of seed accessory structures in angiosperms. We also provide evidence that this pathway likely operates in the nourishing structure of gymnosperm seeds, but rather under maternal genetic control. We therefore propose a model in which the coupling of seed development to fertilization in the angiosperm common ancestors likely coincided with a change in parental expression of auxin biosynthesis genes. This evolutionary innovation likely contributed to the emergence of the endosperm, a defining feature underpinning the rapid diversification and ecological success of flowering plants.

The plesiomorphic seed habit of spermatophytes is the most successful method of sexual reproduction in vascular plants. However, there are key differences between the seeds of its two clades, gymnosperms and angiosperms. In gymnosperm seeds, the embryo is the only biparental structure and it is nourished by a haploid maternal tissue derived from the proliferation of the megagametophyte (Linkies *et al*., 2010; Sakai, 2013). However, in angiosperms, the development of the seed nourishing structure, the endosperm, is tightly linked to fertilization. This is because in angiosperms, both the embryo and the endosperm are biparental, and result from the fertilization of two maternal gametes, the egg cell and the central cell, by two paternal sperm cells. These two fertilization products interact with each other, and the formation of the endosperm is indispensable for embryo viability (Lafon‐Placette & Köhler, 2014). Moreover, these structures are surrounded by a seed coat of maternal sporophytic tissues, which provides various functions (Radchuk & Borisjuk, 2014), and whose development is dependent on signals originating in the biparental endosperm (Weijers *et al*., 2003; Roszak & Köhler, 2011; Figueiredo *et al*., 2016). This coupling to fertilization has obvious advantages, as it prevents the allocation of nutrients to ‘empty seeds’, which do not carry an embryo, and is therefore a major innovation of angiosperms.

Using the model dicot species *Arabidopsis thaliana*, we previously demonstrated that the formation of the biparental endosperm is linked to the paternal expression of auxin biosynthesis genes (Figueiredo *et al*., 2015). This phenomenon, known as genomic imprinting, involves the parent‐of‐origin specific allele expression of certain genes (Batista & Köhler, 2020). The main auxin biosynthesis pathway is a two‐step process, the first being catalyzed by TRYPTOPHAN AMINOTRANSFERASES (TAA/TARs) and the second by YUCCA (YUC) flavin monooxygenases (Mashiguchi *et al*., 2011). Before fertilization, the expression of genes encoding these enzymes is repressed in the maternal gametophyte by H3K27me3 repressive marks (Figueiredo *et al*., 2015; Moreno‐Romero *et al*., 2019), which prevents auxin production in the absence of fertilization. However, the paternal alleles of *TAA/TAR* and *YUC* genes are not labeled with these repressive epigenetic marks and are thus expressed in the fertilized central cell (Figueiredo *et al*., 2015). This means that upon gamete fusion, auxin biosynthesis initiates in a paternal‐dependent fashion, triggering the development of the endosperm and of the surrounding sporophytic tissues (Figueiredo *et al*., 2015, 2016). Consistently, exogenous applications or ectopic production of auxin in unfertilized Arabidopsis ovules lead to the formation of asexual endosperms and seed coats, also known as autonomous seeds (Figueiredo *et al*., 2015, 2016).

Notably, genes involved in auxin biosynthesis are imprinted in the endosperm of other eudicots and of monocots (Luo *et al*., 2011; Waters *et al*., 2013; Hatorangan *et al*., 2016), and auxin biosynthesis seems to be a hallmark of seed nourishing structures (Figueiredo & Köhler, 2018; Florez‐Rueda *et al*., 2024). However, the degree to which these mechanisms are conserved remains to be assessed. Here, we asked if the imprinting of auxin biosynthesis genes is evolutionarily conserved in angiosperms and found that, indeed, auxin drives seed development in early‐diverging angiosperms which diverged *c*. 120–150 million years ago (Ma) from the mesangiosperms (which include the monocot and eudicot clades) (Zuntini *et al*., 2024). We propose that auxin biosynthesis in the seed nourishing structure was likely a feature of the gymnosperm ancestors and became coupled to fertilization in the angiosperm's most recent common ancestor.

## Results and Discussion

**Fig. 1:**
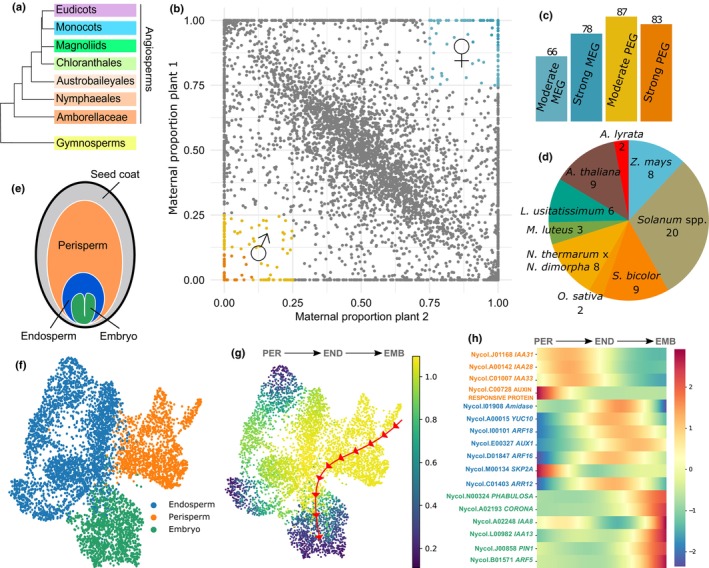
Imprinting and gene expression dynamics in *Nymphaea caerulea* endosperm and seed compartments. (a) Simplified phylogeny of Spermatophytes. (b) Maternal proportions of 5441 genes in reciprocal crosses of *N. caerulea* endosperm. Paternally expressed imprinted genes (PEGs, < 25% maternal proportion) are shown in blue, and maternally expressed genes (MEGs, > 75% maternal proportion) in orange. Darker shades indicate strong PEGs/MEGs (PEGs < 10% or MEGs > 90% maternal proportion). (c) Counts of PEGs and MEGs in *N. caerulea* endosperm, categorized as shown in (b). (d) Number of commonly imprinted genes shared between *N. caerulea* and other angiosperms. (e) Diagram of seed compartments in *Nymphaea*. (f) UMAP visualization of all sequenced nuclei, color‐coded by clusters corresponding to different seed tissues as in (e). (g) Expression trajectory of auxin‐related genes that spans from the perisperm through the endosperm to the embryo. (h) Heatmaps showing dynamic expression of auxin‐related genes along the trajectory outlined in (g). Arrows indicate directionality of transcriptional progression inferred by Palantir pseudotime analysis, with trajectories computed from global gene expression patterns, not restricted to auxin‐related genes. EMB, embryo; END, endosperm; PER: perisperm.

If the mechanisms of seed initiation are conserved in early‐diverging angiosperms and in mesangiosperms, then these mechanisms most likely coupled seed development to fertilization at the origin of angiosperms. To test the hypothesis that auxin biosynthesis is an evolutionarily conserved mechanism for seed initiation, we generated the endosperm imprintome of the early‐diverging water lily species *Nymphaea caerulea* (order Nymphaeales; Fig. 1a, Supporting Information Fig. S1). We used low‐coverage genome sequencing to screen individuals of *N. caerulea* at the Botanical Gardens of Berlin and Potsdam, Germany, and identified two diploid self‐incompatible individuals with a significant level of DNA polymorphisms (henceforth referred to as Plant 1 and Plant 2; Fig. S1; Table S1). We used these individuals for reciprocal crosses and collection of endosperm material using laser capture microdissection; we generated three libraries for each direction of the cross, corresponding to independent pollination events. Sequencing of endosperm‐purified RNA‐seq libraries allowed us to estimate allele‐specific expression for 5441 genes in the *N. caerulea* genome (29% of the total genes expressed in the seed), for both directions of the reciprocal crosses (Fig. 1b; Tables S1, S2). The mean inferred maternal proportions were 0.5192 for Plant 1 and 0.4814 for Plant 2 (Table S1). Very close to 0.5, as expected for a diploid endosperm (Williams & Friedman, 2002). We thus identified 144 candidate maternally expressed genes (MEGs), as those that exceed our imposed threshold of 0.75 maternal proportion in both directions of the cross (Fig. 1b,c; Tables S1, S2). Symmetrically, we inferred 170 paternally expressed genes (PEGs), which show < 0.25 maternal proportion in both cross directions (Fig. 1b,c; Tables S1, S2). Of the 314 imprinted genes identified in the *N. caerulea* endosperm, we found 57 to be imprinted in at least one other species whose endosperm had been examined for imprinting (Fig. 1d; Table S2). Notably, there was an average of seven commonly imprinted genes between species. Such a low number of conserved imprinted genes is expected as imprinting has been shown to evolve rapidly (Hatorangan *et al*., 2016). Interestingly, we found comparatively more PEGs compared to a recent study on *Nymphaea* imprintomes (Povilus *et al*., 2025). However, there are two main differences between the two studies which may account for this discrepancy: (1) our analysis was done in an intraspecific cross, rather than interspecific; and (2) our endosperm samples were collected at an earlier time point. The endosperm in *Nymphaea* seeds is relatively small and devoid of nutrients, and the seed nourishing function is carried out by a maternal sporophytic tissue derived from the ovule nucellus called the perisperm (Fig. 1e; Povilus *et al*., 2015). It is thus reasonable to expect that paternal effects are increasingly reduced as the water lily seed develops, due to the limited role of the endosperm at later stages of development. In such a scenario, PEGs would mostly be detectable in early stages of endosperm development, as shown here, and become less prevalent in later stages of development, as described (Povilus *et al*., 2025). This points to genomic imprinting in the endosperm not being static throughout seed development, but rather a very dynamic phenomenon.

We then asked which gene ontology (GO) terms were enriched in the set of imprinted genes in *N. caerulea* (Fig. S2). We found enriched GO terms pointing to pathways with known roles in seed development, including those involved in epigenetic processes (Table S2). Predicted protein interaction networks can be found in Fig. S2. Notably, genes involved in hormonal metabolism were also found in enriched functional categories, and this included genes involved in auxin biosynthesis, such as *YUC10* (Table S2). In fact, the PEG *YUC10* is one of the only three genes that we found imprinted in endosperms of both *N. caerulea* and in species of monocots and eudicots (Table S2), fitting with our hypothesis that paternal expression of auxin biosynthesis genes is a conserved feature of endosperms. To validate that auxin‐related processes are enriched during seed development in *Nymphaea*, we also performed single‐cell transcriptomics of seed tissues of *N. caerulea* and annotated three distinct clusters as embryo, endosperm and perisperm/seed coat (Fig. 1e,f), based on reference laser‐captured microdissected transcriptomes of the seed tissues (Florez‐Rueda *et al*., 2024; Figs S3, S4; Tables S3, S4). The single‐nucleus analysis, combined with pseudotime inference using Palantir, allowed us to describe transcriptional trajectories across the developing seed, which reflect gradual shifts in global gene expression patterns rather than developmental time or lineage relationships. Palantir identified three such trajectories, all originating from a transcriptionally distinct region within the perisperm cluster and diverging toward different terminal states: one culminating in the embryo (Fig. 1g) and two others in separate domains of the endosperm (Fig. S5). We observed that auxin‐related gene expression aligns closely with the inferred pseudotemporal progression. Heatmaps of auxin‐related genes (Figs 1h, S5) illustrate this pattern more explicitly, suggesting a functional modulation of auxin signaling across distinct seed compartments. For example, the perisperm strongly expressed the auxin‐responsive genes *IAA21*, *IAA28*, and *IAA33*. While the endosperm, where the auxin biosynthesis PEG *YUC10* peaked in expression, also showed elevated expression of key auxin signaling components and associated genes, including *ARF16* and *18*, *AUX1*, and *ARR12*. Within the embryo, crucial auxin signaling genes such as *IAA8* and *13*, *ARF5*, *PIN1*, *PHABULOSA*, and *CORONA* were prominently expressed (Figs 1f, S5, S6; Table S4). This analysis confirms that the expression of *YUC10* is specific to *Nymphaea* endosperms, while highlighting a potential role of auxin in the development of all three of its seed structures. Together, these analyses support the concept of a transcriptionally progressive organization within the seed, where auxin‐related genes exhibit distinct, coordinated expression changes that mirror broader transcriptional trajectories – from the maternally derived perisperm toward the embryo and endosperm compartments, which increasingly specialize in differentiation and assimilation. This is also the first detailed map of the spatial compartmentalization of gene expression at the single‐nucleus level in an early diverging angiosperm.

To test whether water lily seeds accumulate auxin after fertilization, we quantified bioactive auxin, IAA, in somatic and seed tissues of several species of *Nymphaea* (Fig. S7). While early developing seeds showed IAA levels similar to those of somatic tissues, later stages of seed development coincided with an accumulation of auxin (Fig. S7; Table S5). We also confirmed these observations in giant water lilies (*Victoria cruziana* and *Victoria amazonica*; Fig. S7; Table S5), demonstrating that this feature is conserved in the Nymphaeaceae. In fact, auxin accumulation in developing seeds was shown in maize over 80 years ago (Avery *et al*., 1942a,b), and this phenomenon has been confirmed in multiple species, both eudicots and monocots (Teubner, 1953; Lur & Setter, 1993; Abu‐Zaitoon *et al*., 2012; Figueiredo *et al*., 2015; Guo *et al*., 2022), solidifying the argument that post‐fertilization auxin accumulation is a feature of angiosperm seeds.

**Fig. 2:**
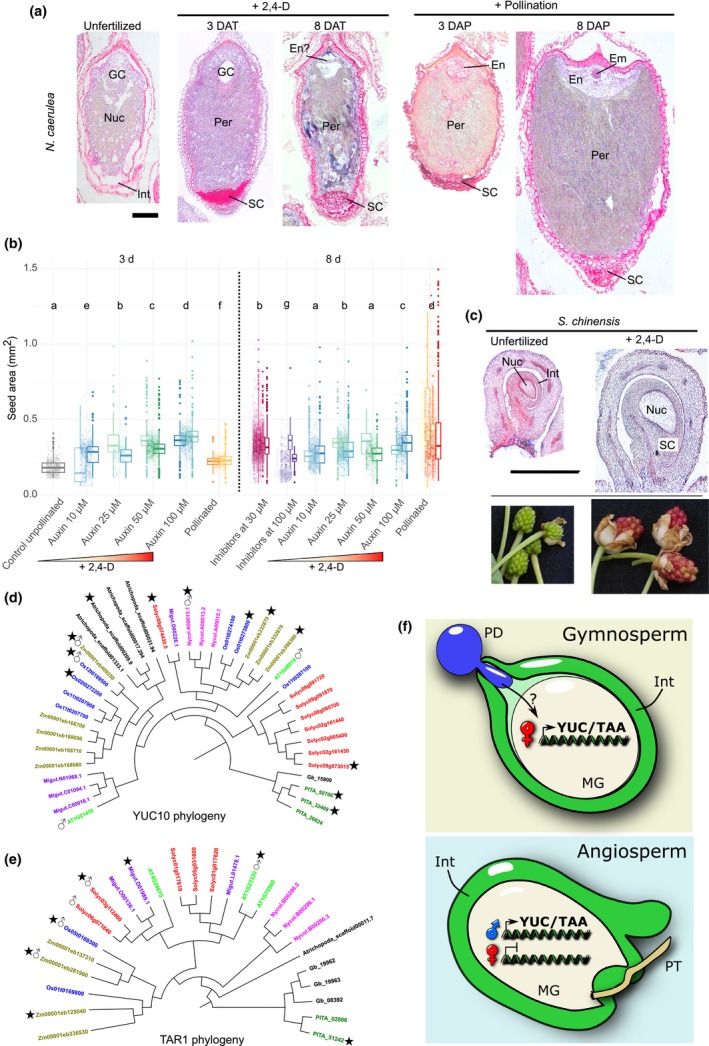
Auxin is an evolutionarily conserved driver of seed development. (a) Left, unfertilized *Nymphaea caerulea* ovule. Middle panels, auxin‐induced autonomous seeds at 3 and 8 d after treatment (DAT). Right panels, fertilized seeds at 3 and 8 d after pollination (DAP). Em, embryo; En, endosperm; GC, gametophytic cavity; Int, integuments; Nuc, nucellus; Per, perisperm; SC, seed coat. Bar, 100 μm. (b) Size of unfertilized ovules, autonomous, and fertilized seeds of *N. caerulea*. Left panel, seed sizes at 3 DAT after application of different concentrations of 2,4‐dichlorophenoxyacetic acid (2,4‐D). Right panel, seed sizes at 8 DAT, after application of different concentrations of 2,4‐D or of the auxin inhibitors yucasin and l‐Kynurenine. Each bar represents one fruit and each dot one seed. The horizontal bar indicates the median, and the error bars indicate SD. Letters indicate Wilcoxon rank‐sum test, *P* < 0.01. (c) Upper panels, *Schisandra chinensis* ovule (left) and auxin‐induced autonomous seed (right) at 10 DAT. Bar, 1 mm. Lower panels, outer ovule morphology in mock and auxin‐treated samples. (d, e) Cladograms of (d) Orthogroups OG0001099 (*YUC* orthologs) and (e) OG0001584 (*TAR* orthologs) denoting genes enriched in the nourishing tissues of the seed. Orthogroup inference and differentially expressed gene (DEG) analyses as inferred previously (Florez‐Rueda *et al*., 2024) and in this study. Imprinted statuses as previously determined (Gehring *et al*., 2011; Hsieh *et al*., 2011; Luo *et al*., 2011; Wolff *et al*., 2011; Zhang *et al*., 2011; Waters *et al*., 2013; Chen *et al*., 2018; Roth *et al*., 2018) and in this study. ★ DEGs ♂ PEGs (paternally expressed genes). Dark green, *Pinus pinaster*; fucsia, *N. caerulea*; bright green, *Arabidopsis thaliana*; red, tomato; purple, *Micrococcus luteus*; blue, *Oryza sativa*; brown, *Zea mays*; black, other species. (f) Proposed model of seed initiation by auxin. In gymnosperms, the deposition of pollen in the pollination drop triggers auxin‐related processes in the ovule. This coincides with the maternal expression of auxin biosynthesis genes in the megagametophyte. In angiosperms, seed development became coupled to fertilization, and thus auxin biosynthesis in the fertilized central cell switched to paternal control. Int, integuments; MG, megagametophyte; PD, pollination drop; PT, pollen tube.

To further challenge our hypothesis that auxin is an evolutionarily conserved driver of seed development, we tested if exogenous application of auxin is sufficient to bypass pollination and lead to fertilization‐independent seed formation in early‐diverging lineages. Indeed, unfertilized *N. caerulea* ovules treated with auxin increase in size, even quicker than their fertilized counterparts (Fig. 2a,b; Table S5), and show signs of tissue differentiation, namely of the sporophytic tissues. This includes the formation of a perisperm and the differentiation of the endothelial seed coat layer (Fig. 2a). However, unlike fertilized seeds, these autonomous seeds are short‐lived and many collapse 8 d after the treatments (8 DAT). A defined endosperm structure is also not often seen, and by 8 DAT only remnants of it are observed (Fig. 2a). We thus took seed size as a marker for auxin‐induced seed formation. Consistent with a role of auxin in driving seed development in *N. caerulea*, auxin applications show a marked dose–response in driving fertilization‐independent ovule enlargement, and the application of inhibitors of auxin biosynthesis (He *et al*., 2011; Kakei *et al*., 2015) blocks the growth of its fertilized seeds in a dose‐dependent manner (Fig. 2b; Table S5). Then, to rule out that this role of auxin is specific to *N. caerulea*, we repeated the auxin applications to four other species of *Nymphaea*, with identical results (Fig. S8; Table S5). The same was true for the giant water lilies of the genus *Victoria* (Fig. S8). Thus, we then expanded our work to *Schisandra chinensis*, which is a self‐incompatible species of liana of the Austrobaileyales clade (Fig. 1a). Again, we observed that auxin induces autonomous seed formation in *S*. *chinensis*, which includes expansion of the nucellus and growth of the surrounding sporophytic structures (Figs 2c, S8). This means that the effect of auxin is not specific to the Nymphaeales but is also true for other early‐diverging angiosperm lineages.

To further assess whether the transcriptional programs triggered by exogenous auxin resemble those activated by fertilization, we compared the transcriptomes of auxin‐treated ovules and fertilized seeds of *N. caerulea* to those of unfertilized/untreated ovules (Fig. S9; Table S6). Fertilized seeds showed a larger set of differentially expressed genes (1097 DEGs) compared to unfertilized ovules, consistent with the activation of endosperm development programs. Auxin‐treated ovules, while morphologically enlarged, activated fewer DEGs (716), in line with their failure to establish an endosperm. Nevertheless, 129 genes were commonly upregulated in both auxin‐treated ovules and in fertilized seeds (odds ratio = 4.2, Fisher's *P* = 7.5 × 10^−35^), indicating partial activation of fertilization‐associated pathways (Fig. S9; Table S6). This overlap included multiple components of auxin biosynthesis, signaling, and transport – such as *GH3.1*, *IAA31*, *ARF5*, *ARF16*, and *SAUR31* – as well as transporters (*ABC* transporters, *SWEET7*), transcription factors (*bZIP44*, *ERF4*, *GTL1*, *TGA9*, and *ZHD9*), and genes linked to seed coat and perisperm differentiation (*PDF1*, *LEA*). Several of these genes, including *ARF5, ARF16, GTL1, IAA31*, and *SWEET7*, among others, were also recovered in the top‐ranked genes per tissue of our single‐cell transcriptome dataset of fertilized seeds (Table S4; Fig. 1h). These findings support that exogenous auxin partially recapitulates the molecular programs normally initiated by fertilization, particularly those related to auxin signaling and sporophytic tissue differentiation.

Thus far, our data points to post‐fertilization auxin biosynthesis in the endosperm being a conserved driver of seed development in the angiosperms. This is supported by the observation that auxin biosynthesis genes are endosperm PEGs in *Nymphaea* (this study) and in species from which the Nymphaeales diverged over 100 Ma (Hsieh *et al*., 2011; Wolff *et al*., 2011; Zhang *et al*., 2011; Waters *et al*., 2013; Pignatta *et al*., 2014; Figueiredo *et al*., 2015; Chen *et al*., 2018; Roth *et al*., 2018). This is particularly striking as there is limited conservation in imprinting even among closely related lineages (Hatorangan *et al*., 2016; Klosinska *et al*., 2016), and auxin biosynthesis genes are thus the exception to that trend. We thus postulated that those genes should have a common evolutionary origin and belong to the same orthogroups (OGs) (Emms & Kelly, 2019). Based on our previous comparative transcriptomic dataset of seed nourishing tissues (Florez‐Rueda *et al*., 2024), we identified two OGs, OG0001099 and OG0001584, which contain the land plant *YUC10* and *TAR1* orthologs, respectively (Table S7). Fitting with our hypothesis, several members of these OGs are specifically expressed in the endosperm of each angiosperm species and form a clade (indicated by stars in Fig. 2d,e; Vijayanathan *et al*., 2025). Remarkably, several genes belonging to these OGs have already been described as PEGs in angiosperm endosperms (indicated by male symbols in Fig. 2d,e). Importantly, our previous study of early‐diverging angiosperm transcriptomes also supports an ancestral role for auxin in angiosperm endosperm development in those taxa (Florez‐Rueda *et al*., 2024): in *Amborella trichopoda* and *N. caerulea*, the *YUC10* orthologs *Atrichopoda_scaffold00209.9* and *Nycol.A00013.1* are specifically expressed in their endosperms, and not in other seed or vegetative tissues (Fig. 2d,e; Table S7).

As alluded to above, the double fertilization of the angiosperm megagametophyte is an innovation of the angiosperms, leading to the formation of the biparental embryo and endosperm. However, in gymnosperms, the seed nourishing function is provided by the haploid maternal megagametophyte, which develops independently of fertilization. Strikingly, the development of the mature ovule in the gymnosperm *Ginkgo biloba* L., which includes the maturation of the megagametophyte, is tightly linked to auxin activity (Muto *et al*., 2024). However, unlike in angiosperms where auxin responses are triggered by fertilization, in *G. biloba*, these processes are linked rather to pollen deposition in the ovule (Muto *et al*., 2024). We thus hypothesized that the development of the megagametophyte into a nourishing structure should be linked to the expression of auxin biosynthesis genes in both lineages of spermatophytes, even if its parent‐of‐origin would be different: maternal in the gymnosperm megagametophyte, and paternal in the angiosperm endosperm. If that is true, then orthologs of the angiosperm endosperm auxin biosynthesis PEGs should be expressed in the gymnosperm megagametophytes. To test this hypothesis, we mined our previously published transcriptomic profiles of maritime pine (*Pinus pinaster*) megagametophytes, collected post‐pollination (Florez‐Rueda *et al*., 2024), and found that this indeed was the case: the *P. pinaster* megagametophyte expresses the *TAR1* homolog *PITA_50706* and two *YUC10* homologs, *PITA_32408* and *PITA_5142* (Fig. 2d,e; Table S7). As predicted, those genes fall into the same OGs as their angiosperm PEG counterparts, OG0001099 and OG0001584 (Table S7), and belong to the same *YUC* and *TAA/TAR* clades (Vijayanathan *et al*., 2025). Our observations allow us to conclude that auxin production is a feature of at least some gymnosperm megagametophytes, suggesting that a role for auxin in the development of the nutritive tissues of the seed predates the emergence of the angiosperm clade and the origin of the endosperm proper. Because the gymnosperm seed‐nourishing structure is purely of maternal origin, the expression of auxin biosynthesis genes would be maternal gametophytic in the gymnosperms, and that the parent‐of‐origin expression control shifted to the father in the angiosperms. These genes thus likely became PEGs after the evolution of double fertilization in the angiosperm common ancestor. However, although the expression of auxin biosynthesis genes is of maternal origin in the gymnosperm seed nourishing structure, we cannot rule out that germinating pollen and the resulting tube could also be involved in auxin production. Uncovering what the signals are that originate in the pollen and trigger the development of the nourishing structure will be an exciting area of future research.

We thus propose a model where auxin is an evolutionarily conserved driver of seed development in spermatophytes (Fig. 2f): in gymnosperms, the maternal expression of auxin biosynthesis genes in the nourishing seed structure is seemingly triggered by pollination, by so far unknown mechanisms, while in angiosperms auxin biosynthesis in the endosperm is paternal in nature, coupling the development of the seed nourishing structure and accessory tissues to fertilization. This innovative switch in control likely ensures a tighter post‐fertilization biparental control over resource allocation to the seeds, thus contributing to the emergence of the endosperm as a pivotal innovation that underpinned the evolutionary success of flowering plants.

## Materials and Methods

### Plant materials

*Nymphaea* plants were screened in the Botanical Gardens of Potsdam and Berlin, Germany, in the summer of 2021 to identify self‐incompatible taxa. Several species were screened for diploidy and self‐incompatibility. Ploidy was estimated by comparing the genome size (pg/2C) obtained via flow cytometry to published genome size values of diploid and polyploid species within the genus (Pellicer *et al*., 2013). Controlled crosses, emasculations, and continuous observations led us to select two polymorphic individuals of the species *Nymphaea caerulea* (Savigny) Verdc. maintained in the living collections of the Botanical Garden of Potsdam. Flowers were bagged before anthesis, and seed production was null or low, which is indicative of self‐incompatibility. This was in strong contrast to other species of the genus which develop seeds even after emasculations on the day of female receptibility. *Nymphaea caerulea* was one of the few *Nymphaea* species that we analyzed where emasculation easily prevented self‐fertilization and in which events of self‐fertilized seeds were rare in the glasshouse conditions in Potsdam.

Since we aimed to detect imprinting, an important criterion was the levels of polymorphism between accessions; we aimed to have at least two individuals that had polymorphisms between them that allowed for allelic specific expression analyses and imprinting inference. We therefore screened three individuals of *Nymphaea colorata* (Casp.) Verdc. and two of *N. caerulea* from the Berlin and Potsdam Botanical Gardens. We collected leaf material and extracted DNA, which was used for genome resequencing on the DNBseq platform (BGI, Shenzhen, China) with 150 bp paired‐end reads. We then assessed the number of polymorphic sites between the resequenced genomes (Fig. S1; Table S1). These genome resequencing datasets were used for imprinting inference, as described below. Experiments on *Schisandra chinensis* (Turcz.) Baill. and *Victoria* sp. were performed using individuals at the Berlin and Potsdam Botanical Gardens.

### Hormonal treatments and comparative gene expression analysis

To test the effect of auxin as an inducer of seed development, we treated a number of Nymphaeales species with 2,4‐dichlorophenoxyacetic acid (2,4‐D), a synthetic auxin. The experiments were carried out in the botanical gardens of Berlin and Potsdam. During the spring–summer season, flowers of different Nymphaeales species, namely *N. caerulea*, *N. daubenyana* O.Thomas, *N. nouchali* Burm. f., *N. alba* L., *V. amazonica* (Poepp.) J.C. Sowerby, and *V. cruziana* A.D.Orb., were used for the experiments. We also used a sterile hybrid accession that flowers profusely but never develops seeds after pollination. This accession is called *Nymphaea* × *purple hybrid*.

For the experiments, we identified flowers on their first day of opening when they were female receptive, a state characterized by the accumulation of fluid in the carpels. For negative control experiments, we used female receptive plants and carefully removed their anthers, which should be pollen‐free and bagged the flowers to prevent pollination in the glasshouse. For pollination experiments, the flowers were pollinated on female receptive days as a positive control. For the auxin application experiments, flowers were emasculated and then 100 μM 2,4‐D was applied to the carpel fluid on female receptive days. The volume of 2,4‐D solution was adapted to the size of the flower. For example, up to 5 ml to fill the large carpel cup of *Victoria* spp. flowers to a minimum of 100 μl to fill the small carpel cup of *N. alba* flowers.

Further experiments were carried out at the Botanic Garden in Berlin on plants of *S. chinensis* as another representative of an early divergent angiosperm clade, Austrobaileyales. As *S. chinensis* plants are dioecious and only one female individual exists in the collection of the Berlin Botanical Garden, only control and 2,4‐D experiments were carried out. This was also the case for the flowers of the *Victoria* spp., as pollinations were not successful in any of our experiments in 2021. Similarly, our pollination experiments with the *Nymphaea* × *purple hybrid* failed to produce viable seeds.

For all experiments in the season of 2021, flowers were collected a week after anthesis and immediately fixed by vacuum infiltration in farmer's fixative (9 : 1, ethanol : acetic acid). The material was kept in the fixative until further processing as described below.

A set of *N. caerulea* plants was then established in the glasshouse of the Max Planck Institute of Molecular Plant Physiology, Potsdam‐Golm, in 2022. Further experiments were carried out on these two plants throughout 2022 and 2023 (referred to as Plants 1 and 2). We expanded our sample collections to an earlier time point, 3 d after anthesis (DAA), and also continued our experiments a week after anthesis. With the aim of identifying a threshold for the effect of auxin, we performed a gradient experiment of increasing auxin concentration from 10 to 100 μM 2,4‐D. In addition, to test the effects of auxin depletion in the seed, we performed exogenous applications of the auxin inhibitors l‐Kynurenine (He *et al*., 2011) and Yucasin (Kakei *et al*., 2015) to the carpel fluid. We tested two concentrations of each chemical, 30 and 100 μM. In brief, plants were manually pollinated on the morning of the first flower opening, and at least 3 h later, each inhibitor was applied to the carpel cup. For this set of experiments, whole flowers were collected and frozen at −80°C. A piece of the carpel was fixed and processed for histological imaging as described below. For seed size measurements, the seeds were manually dissected from their embedding tissue and carefully separated to allow seed area measurements. Seeds or ovules were placed in Petri dishes with cold PBS 1×, imaged and their area immediately measured using a Keyence digital microscope. For all hormonal treatments, mock controls were run in parallel. Seed size differences between treatments were assessed using the Wilcoxon rank‐sum test on data aggregated by treatment from individual fruits (biological replicates). Analyses were performed in R, and results are provided in Table S5.

To further investigate whether the developmental programs triggered by auxin resemble those activated by fertilization, we performed a comparative transcriptomic analysis on *N. caerulea*. Samples were collected from flowers at anthesis (0 DAA), a week after hand‐pollination, and a week after the application of 100 μM 2,4‐D, following the treatment procedures described above. For each condition, three biological replicates were obtained from whole seeds, which were manually dissected from the fruit tissue, washed, and immediately frozen in liquid nitrogen. Total RNA was extracted using standard protocols and shipped to Novogene (Munich, Germany) for library preparation and sequencing. Libraries were prepared using Illumina TruSeq protocols and sequenced on an Illumina NovaSeq 6000 platform (Illumina Inc., San Diego, CA, USA). Details of the datasets generated are provided in Table S8.

### Cytological observations

Fruits were manually sectioned to allow embedding in paraffin blocks. The blocks were sectioned at 7 μm on a microtome. Paraffin ribbons were placed on microscope slides, which were further prepared and stained as follows. Slides were dewaxed by immersion in xylol or histoclear twice for 10 min. Sections were hydrated through a graded ethanol series to 100% water. For general tissue structure, sections were stained with 1% Safranin for 5 min and with Aniline Blue 2% in 3% acetic acid for 1 min. After a brief water wash, the slides were passed rapidly through a graded ethanol series up to 100% ethanol, taking care to preserve the staining. Finally, the slides were mounted in Roti Mount Aqua. Images were captured on an Olympus motorized epi‐fluorescence microscope using CellSens software, and seed area was measured by manually demarcating the most central section of individual seeds using Fiji.

### Compartment‐specific transcriptome generation and LCM

As part of the large set of experiments of 2021, pollinated flowers of *N. caerulea* at 1 wk after pollination were selected for targeted Laser‐capture microdissection (LCM) experiments. We aimed to produce transcriptomes of the main compartments of the Nymphaea seed: endosperm, embryo, and perisperm. We used three different flowers numbered 47, 105, and 115. Flowers were collected from the plants of the Botanical Garden Potsdam a week after pollination. These plants were later established on the Max Planck Institute of Molecular Plant Physiology (MPIMP) in 2022 and used for imprinting inference (to be described later). LCM was performed as follows. Developing fruits were harvested a week after manual pollination. Material was immediately immersed in cold farmers' fixative (ethanol : acetic acid solution, 9 : 1) and vacuum infiltrated. The material was kept in the fixative until embedding which took place in less than a month after collection. Fruits were manually sectioned to allow accommodation in the paraffin blocks. Blocks were sectioned on a microtome at 7 μm. LCM protocols were followed as previously described (Florez‐Rueda *et al*., 2016a). In brief, capture was performed using a Leica LCM microscope carefully separating the endosperm from the embryo and surrounding perisperm and seed coat tissue. Target tissue was laser captured using a Leica LMD600 microscope at the MPIMP and RNA extraction was performed immediately or within 24 h; in the latter case, the caps were stored at −80°C before extraction. RNA extraction was performed using the Applied Biosystems^®^ Arcturus^®^PicoPure^®^ RNA Isolation Kit (ref. KIT0204; Thermo Fisher Scientific, Waltham, MA, USA) according to the manufacturer's instructions. The quality and quantity of total RNA were assessed with Bioanalyzer Pico Chips (Agilent, Santa Clara, CA, USA). RNA that showed clear ribosomal peaks was used for further steps. From the extracted RNA, cDNA was amplified using the SMART‐Seq^®^ v.4 Ultra^®^ Low Input RNA Kit for Sequencing (Takara Bio, San Jose, CA, USA). cDNA amplification was validated by running Agilent 2100 Bioanalyzer High Sensitivity DNA Chip (Agilent). After validating successful library preparation with fragment sizes ranging from 400 to 600 bp, libraries were sequenced with 150 bp paired‐end reads on an Illumina NovaSeq 6000 (Illumina Inc.) platform at Novogene in the UK. Details of the datasets generated are in Table S8.

### Crossing experiments for imprinting inference

Reciprocal crosses were performed on the two polymorphic individuals of *N. caerulea* established in the MPIMP during the summer of 2022. Three different flowers per direction of the cross were used for the study and were collected 1 wk after controlled pollination. For these six samples, endosperm tissue was laser‐captured and its transcriptome was generated as described above. Parental genomes were previously sequenced at the Beijing Genomics Institute to guide the selection of appropriate individuals for this experiment (see ‘Plant materials’ in the Materials and Methods section).

### Bioinformatic analyses of LCM data for imprinting inference

Quality of the reads was assessed with FastQC. Raw reads were filtered by quality, adapters removed, and trimmed with Trimmomatic using the parameters: LEADING:3 TRAILING:3 MINLEN:50 HEADCROP:20. We used a Snakemake pipeline to perform further bioinformatic analyses, from mapping up to maternal proportions per gene (https://github.com/mscharmann/ASAP). Mapping of the reads was done against the *N. colorata* v.1.2 genome (available at https://data.jgi.doe.gov/refine‐download/phytozome?organism=Ncolorata&expanded=566) using Burrows–Wheeler Aligner. We generated three independent endosperm libraries per cross direction and, after separate mapping, merged the resulting BAM files before variant calling to capture consistent allele‐specific expression biases in the endosperm. Variant calling was performed with Vcftools (Danecek *et al*., 2011). We filtered variants to retain only high‐confidence, bi‐allelic sites with a minor allele count ≥ 2, quality ≥ 20 and depth ≥ 5. Our allele‐specific expression pipeline to detect imprinting informative sites in the endosperm followed the rationale to not only make use of reciprocal homozygous sites but also of heterozygous sites in either parent as implemented before for the tomato (*Solanum lycopersicum* L.) imprintome (Florez‐Rueda *et al*., 2016b). In brief, variant sites between the parental plants were recovered using genome resequencing of both parental individuals. Using a custom Python program, we transformed the frequencies of the discriminant alleles in each single nucleotide polymorphism (SNP) into maternal proportions per gene in each direction of the cross. Once a per gene value was obtained for each direction of the cross, we used thresholds for maternal proportions in reciprocal crosses to call a given gene as potentially imprinted and considered moderately and strongly imprinted genes, as follows: moderate MEGs > 0.75, strong MEGs > 0.9, moderate PEGs < 0.25, and strong PEGs < 0.1 maternal proportion. These thresholds are reciprocally symmetric and consistent with the expectation of a 0.5 maternal proportion of gene expression in the diploid endosperm of *Nymphaea*. Furthermore, genes considered candidate MEGs and PEGs exhibited significant departures from the expected 0.5, as assessed by chi‐squared tests with false discovery rate (FDR) corrections. A full list of *N. caerulea* imprinted genes with summary statistics can be found in Table S2, and details of the inference pipeline in Table S1.

Lists of imprinted genes in other taxa were checked for overlap with the list of imprinted genes in the *N. caerulea* endosperm reported here. Lists from *A. thaliana* (L.) Heynh. (Gehring *et al*., 2011; Hsieh *et al*., 2011), *A. lyrata* (L.) O'Kane & Al‐Shehbaz (Klosinska *et al*., 2016), *Zea mays* L. (Waters *et al*., 2013), *Oryza sativa* L. (Chen *et al*., 2018), *Sorghum bicolor* (L.) Moench (Zhang *et al*., 2016), *Linum usitatissimum* L. (Jiang *et al*., 2021), *Micrococcus luteus* (L.) G.L.Nesom (Kinser *et al*., 2021), species of wild tomatoes *Solanum* spp. L. (Roth *et al*., 2018), and an interspecific cross of the water lilies *Nymphaea thermarum* Eb.Fisch. and *Nymphaea dimorpha* I.M.Turner (Povilus *et al*., 2025) were collated and checked for overlap.

### Differential gene expression analyses of compartment‐specific and hormonal treatment transcriptomes

All RNA‐seq datasets generated in this study were processed using the same mapping, quantification, and enrichment analysis pipelines, with differential expression models adapted to each experimental design. For all analyses, reads were mapped to the *N. colorata* v.1.2 genome (available at https://data.jgi.doe.gov/refine‐download/phytozome?organism=Ncolorata&expanded=566) using Hisat2 (Kim *et al*., 2019). Transcript assembly and quantification were performed with Stringtie (Pertea *et al*., 2015), and read count matrices were generated for downstream statistical analyses. Genes with fewer than 10 reads in at least two samples were excluded.

For the compartment‐specific LCM transcriptome analyses, differential gene expression tests were performed using a compound factor (tissue) with multiple levels (sample ID) using DESeq2 (Love *et al*., 2014) in R. For these analyses, the endosperm libraries generated for imprinting inference in 2022 were also included in the analyses. We tested for differential expression between pairs of seed compartments, namely Endosperm and Embryo, Endosperm and Perisperm, and Embryo and Perisperm. DEGs were defined as those with FDR‐corrected *P*‐values < 0.05 and absolute fold changes > 2. To delimit a set of genes preferentially expressed in each of the major seed organs, embryo, endosperm, and perisperm, we identified the top set of genes expressed in a given tissue as the intersection of those significantly upregulated in both comparisons including the tissue. For visualization, Venn diagrams were generated using the package ggvenndiagram (Gao *et al*., 2021) in R. Full supplementary tables of the total DEGs per comparison and the top sets of DEGs in each tissue are provided in Table S3. Fig. S10 shows a PCA plot of the LCM datasets, including those used for imprinting inference, showing tissue‐specific clustering along PC4.

For the hormonal treatment experiment on *N. caerulea* seeds (0 DAA, a week after fertilization, and a week after auxin treatment), pairwise comparisons were made between unfertilized vs fertilized seeds and unfertilized vs auxin‐treated seeds in R (Love *et al*., 2014). DEGs were defined using the same significance criteria as above (FDR‐adjusted *P*‐value < 0.05, absolute fold change > 2). To identify shared transcriptional programs between auxin‐treated ovules and fertilized seeds, the overlap in DEG sets was determined, and its statistical significance was assessed by a hypergeometric test in R. Full DEG lists for both comparisons, enrichment analyses and overlap are provided in Table S6.

### Enrichment and visualization

For all DEG sets from all experiments, functional enrichment was carried out in STRING (Szklarczyk *et al*., 2019) and Panther (Mi *et al*., 2019) using the respective expressed gene sets as the background (18 962 genes for the LCM compartment experiments and 20 327 genes for the hormonal treatment experiments). All reported enriched terms had FDR‐adjusted *P*‐values < 0.05. To further classify genes into functional modules, the MCL clustering algorithm in STRING was applied with an inflation parameter of 1.5. Heat maps were generated with the complexheatmap package in R to visualize expression patterns of selected gene sets (Gu, 2022).

### Nuclei extraction for single cell RNA‐sequencing

To further investigate the seeds of *N. caerulea*, we conducted single‐cell transcriptomic analysis of seed compartments. Seeds were collected 1 wk after manual pollination, by which time they had turned red, indicating successful pollination. We removed the outer red cell layer and dissected the seeds to discard excess starch from the outer perisperm layers and to enrich the smaller endosperm and embryo. The endosperms, embryos, and surrounding perisperm tissue were then isolated. Up to 150 seeds were manually dissected with at least four washes in fresh, cold PBS 1% to eliminate debris. Nuclei were extracted from the dissected tissue using the CyStain™ UV Precise P kit, following the manufacturer's protocol with minor modifications. All subsequent steps were performed readily, on ice, and reagents were kept cold. The nuclei extraction buffer was supplemented with protease inhibitors, 2% BSA, and RNAse inhibitors (Ambion RNase Inhibitor, SUPERaseIn™ RNase Inhibitor, and RiboLock). The tissue was macerated in the supplemented nuclei extraction buffer for no less than 2 min and immediately filtered through a 30 μm filter. This was followed by centrifugation at 500 ***g*** for 5 min at 4°C. After removing the supernatant, 500 μl of staining buffer was added, and the nuclei were gently resuspended before passing through a 10 μm filter. A second centrifugation was performed, and the nuclear solution was reduced to 100 μl. Nuclei were counted using a Neubauer chamber. Library preparation was conducted using 10× Genomics kits according to the manufacturer's instructions, and sequencing was performed by Novogene. Three libraries were successfully generated from the manually dissected *N. caerulea* seeds.

### Bioinformatic analyses of single‐cell RNA‐seq data

Raw sequencing data were processed using Salmon and Alevin‐fry (He *et al*., 2022) to generate gene‐level count matrices. Quality control and filtering were conducted with Scampy (Wolf *et al*., 2018). To address cell‐free RNA contamination, we utilized EmptyDrops (Lun *et al*., 2019) and further corrected for ambient RNA with SoupX (Young & Behjati, 2020). scDblFinder was employed to remove doublets (Germain *et al*., 2022). Data from three libraries were integrated using Harmony to correct for batch effects (Korsunsky *et al*., 2019). Clustering was performed with the Leiden algorithm (resolution 0.01), yielding three clusters. Top‐ranked marker genes for each cluster were identified (Table S4: Cluster Description). Functional enrichment and network analyses of the top‐ranked genes per cluster were conducted using STRING (Szklarczyk *et al*., 2017). Gene universe comprised all the genes detected in this dataset. Pseudotime and cell trajectory inferences were made using Palantir (Setty *et al*., 2019) to explore dynamic gene expression changes and lineage relationships.

### Metabolomic experiments

Collection of seeds for hormone measurements through mass spectrometry (MS) was performed in the Botanical Gardens of Potsdam and Berlin. For the genus *Victoria*, two plants of *V. cruziana* were used for the experiments, one in Potsdam and one in Berlin. Plants were cross‐pollinated, but the success of the crossings was very low. Successful pollinations were evident because of the increased size of the seeds as they developed. Flowers were collected at an early time point, up to 3 d after they had opened and when their receptivity was finished. A later time point at 2 wk was achieved only twice and only a few seeds were collected for MS measurements. Seeds were carefully dissected and immediately flash frozen and stored for processing at −80°C. Additionally, leaf and root tissue were sampled. Data points correspond to single seeds in each of the plants used.

For the *Nymphaea* seed hormone measurements, a wide array of species of the Nymphaea Section Brachyceras from the Botanical Garden Berlin was used for experiments in 2021. Most accessions available were self‐compatible and developed seeds without the need for manual pollination, but manual pollinations were implemented on flowers destined to be used for measurements. Visits were performed weekly and three time points of collection were defined: first, an early time point being up to 3 DAA; second, a week after controlled pollination, characterized by a red color in the seeds in most of the species studied; and third, a later time point at 3 wk after pollination, a time point in which the seeds are reaching maturity and are colored black or dark brown. Seeds or leaf tissue were dissected and flash frozen and stored for processing at −80°C. Data points refer to independent replicates of pooled seeds per species.

We implemented a liquid : liquid metabolite extraction protocol that allowed for the qualitative analyses of auxin and some of its conjugates (Salem *et al*., 2020). In brief, extraction was performed using a methyl‐tert‐butyl‐ether (MTBE) : methanol (MeOH) solution. The use of acidified water enabled the purification of the phytohormones into the organic MTBE : MeOH phase for further quantification through Ultra‐High‐Performance Liquid Chromatography (UHPLC)–MS/MS. The quantification of auxin and some of its conjugates was performed with a previously described UHPLC–MS/MS‐targeted method (Salem *et al*., 2020). In brief, extraction of the samples was performed using 1 ml of an MTBE : MeOH solution (3 : 1, v/v) followed by a liquid : liquid purification step with 0.5 ml of water acidified with 0.1% HCl. An aliquot of 500 μl of the upper organic phase containing the phytohormones was dried and resuspended in 50 μl of MeOH : H_2_O (1 : 1, v/v). The samples were analyzed in a UHPLC–MS/MS system consisting of a Waters^®^ (Milford, MA, USA) nanoAcquity UPLC^®^ and a Sciex^®^ (Concord, ON, Canada) linear ion trap 4000 QTRAP^®^. The chromatographic stationary phase consisted of a C18‐column (HSS T3 100 mm × 2.1 mm, 1.8 μm diameter particles; Waters^®^) fitted with a 2.1 × 5 mm guard column (ACQUITY UPLC HSS T3 VanGuard Pre‐column, 100 Å, 1.8 μm; Waters^®^). The mobile phase consisted of a binary solvent system of water containing 0.1% (v/v) formic acid (solvent A) and methanol containing 0.1% (v/v) formic acid (solvent B). The elution gradient was as follows: 62% eluent A for 6.5 min; 45% eluent B from 6.5 to 7.0 min; 10% eluent A from 7.0 to 7.1 min, held at 0% eluent A from 7.1 to 8.1 min; and returned to initial conditions by 8.1 min. From 8.1 to 10.0 min, the column was re‐equilibrated and conditioned to 62% eluent A. The ionization was achieved using electrospray ionization through a Sciex^®^ Turbo V^®^ Ion Source operating in negative ionization mode. The mass spectrometer analyzer was operated using a multiple reaction monitoring method, for the targeted quantification of IAA. The ion transitions (Q1/Q3 Masses: 174.05/130.20 Da) were previously optimized using the authentic standard of IAA. All data processing was performed in the software Analyst 1.6.2 (Sciex^®^). The relative quantification of IAA across samples was performed based on the total peak area for this compound in each sample. Statistical analyses were performed in R, and results are provided in Table S5.

## Competing interests

None declared.

## Author contributions

AMF‐R, JBB, and DDF designed the experiments. AMF‐R performed the experiments. LPdS and ARF performed the metabolomics analyses. MS and AMF‐R coded the imprinting inference pipeline. AMF‐R performed the bioinformatic analyses. AMF‐R and DDF wrote the manuscript with input from all co‐authors.

## Supporting information


**Fig. S1** *Nymphaea* individuals used in this study.
**Fig. S2** Enriched terms and related putative interaction networks for imprinted genes in *Nymphaea caerulea*.
**Fig. S3** Delimitation of tissue‐specific genes based on the intersection of differential expression in pairwise tissue comparisons.
**Fig. S4** Manual annotation of cell clusters based on LCM expression patterns.
**Fig. S5** Single‐cell trajectory and pseudotime analysis of gene expression in the seed compartments of *Nymphaea caerulea*, focusing on auxin‐related genes.
**Fig. S6** Expression of auxin related genes at the single cell resolution in the *Nymphaea caerulea* seed.
**Fig. S7** Auxin is produced after fertilization in Nymphaeales seeds.
**Fig. S8** Auxin experiments in an array of Nymphaeales including species of *Victoria* spp. and in the austrobaileyale *Schisandra chinensis*.
**Fig. S9** Shared gene expression in auxin‐treated and pollinated tissues.
**Fig. S10** PCA plot of LCM RNA‐seq datasets.

**Table S1** Allele‐specific expression pipeline statistics.
**Table S2** Overview of imprinted genes and enrichment.
**Table S3** Seed compartment DGE intersections and enrichment.
**Table S4** Top‐ranked single‐cell gene clusters and overlaps with seed compartment DGE intersections.
**Table S5** Raw data and statistics of auxin‐related experiments.
**Table S6** Hormonal treatment DGE: auxin vs pollination.
**Table S7** Auxin biosynthesis genes and ortholog expression.
**Table S8** Summary of NGS datasets.
Please note: Wiley is not responsible for the content or functionality of any Supporting Information supplied by the authors. Any queries (other than missing material) should be directed to the *New Phytologist* Central Office.

## Data Availability

All data generated is available at NCBI under reference PRJNA1157574.
