# Supplementary material for: Genomic imprinting in an early‐diverging angiosperm reveals an ancient mechanism for seed initiation in flowering plants
Source: New Phytol. 2025 Nov 28;249(4):1580–91. doi: 10.1111/nph.70776 (PMC12825398; doi:10.1111/nph.70776)
Supplement: Supplementary file 1 — Fig. S1 Nymphaea individuals used in this study. Fig. S2 Enriched terms and related putative interaction networks for imprinted genes in Nymphaea caerulea. Fig. S3 Delimitation of tissue‐specific genes based on the intersection of differential expression in pairwise tissue comparisons. Fig. S4 Manual annotation of cell clusters based on LCM expression patterns. Fig. S5 Single‐cell trajectory and pseudotime analysis of gene expression in the seed compartments of Nymphaea caerulea, focusing on auxin‐related genes. Fig. S6 Expression of auxin related genes at the single cell resolution in the Nymphaea caerulea seed. Fig. S7 Auxin is produced after fertilization in Nymphaeales seeds. Fig. S8 Auxin experiments in an array of Nymphaeales including species of Victoria spp. and in the austrobaileyale Schisandra chinensis. Fig. S9 Shared gene expression in auxin‐treated and pollinated tissues. Fig. S10 PCA plot of LCM RNA‐seq datasets. [file NPH-249-1580-s002.docx]

## *New Phytologist* Supporting Information

Article title: Genomic imprinting in an early-diverging angiosperm reveals an ancient mechanism for seed initiation in flowering plants

Authors: Ana M. Florez-Rueda, Mathias Scharmann, Leonardo P. de Souza, Alisdair R. Fernie, Julien B. Bachelier, Duarte D. Figueiredo

Article acceptance date: 31 October 2025

The following Supporting Information is available for this article:

**Fig. S1** *Nymphaea* individuals used in this study.

**Fig. S2** Enriched terms and related putative interaction networks for imprinted genes in *N. caerulea*.

**Fig. S3** Delimitation of tissue-specific genes based on the intersection of differential expression in pairwise tissue comparisons.

**Fig. S4** Manual annotation of cell clusters based on LCM expression patterns.

**Fig. S5** Single-cell trajectory and pseudotime analysis of gene expression in the seed compartments of *N. caerulea*, focusing on auxin-related genes.

**Fig. S6** Expression of auxin related genes at the single cell resolution in the *N. caerulea* seed.

**Fig. S7** Auxin is produced after fertilization in Nymphaeales seeds.

**Fig. S8** Auxin experiments in an array of Nymphaeales including species of *Victoria* spp. and in the austrobaileyale *S. chinensis*.

**Fig. S9** Shared gene expression in auxin-treated and pollinated tissues.

**Fig. S10** PCA plots of RNAseq LCM datasets.

**Table S1** Allele-Specific Expression Pipeline Statistics

**Table S2** Overview of Imprinted Genes and Enrichment

**Table S3** Seed Compartment DGE Intersections and Enrichment

**Table S4** Top-Ranked Single-Cell Gene Clusters and Overlaps with Seed Compartment DGE Intersections

**Table S5** Raw Data and Statistics of Auxin-Related Experiments

**Table S6** Hormonal Treatment DGE: Auxin vs. Pollination

**Table S7** Auxin Biosynthesis Genes and Ortholog Expression

**Table S8** Summary of NGS Datasets

**Fig. S1 *Nymphaea* individuals used in this study**. **a**, Representative photo of a *N. careulea* individual used in this study. **b,** Number of informative sites identified in plants 1 and 2 (here called Genotypes A and B). Informative sites being SNPs that can be used to differentiate the two genotypes. Genotypes C and D indicate individuals of *Nymphaea colorata* for which there were almost no informative sites. Such individuals are likely clones and are therefore not useful for studies of genomic imprinting. Hom1_het2, sites homozygous in first genotype and heterozygous in the second, het1_hom2, sites heterozygous in first genotype and homozygous in the second, recipr_hom, sites homozygous in both genotypes.


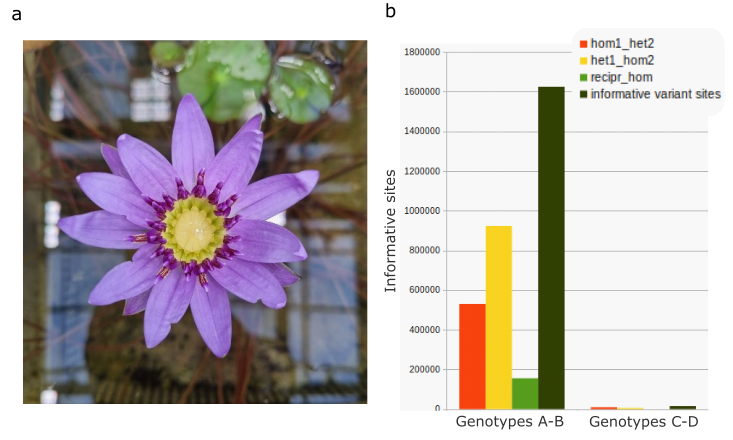


**Fig. S2 Enriched terms and related putative interaction networks for imprinted genes in *N. caerulea*.** **a**, Enriched GO categories among genes imprinted in the endosperm of *N. caerulea*. **b**, Gibberellin-related cluster, containing *GIBBERELLIN 20 OXIDASE 2* (*GA20OX2*), *GIBBERELLIN 2-OXIDASE 8* (*GA2OX8*) and *GIBBERELLIC ACID INSENSITIVE* (*GAI*). **c**, Cluster formed by terms “DNA methylation” (GO:0006306; FDR pval 0.0233), “DNA replication” (KW-0235 FDR pval 0.0212) and “DNA repair” (KW-0234 FDR pval 0.0182). These include genes such as: *FLAP ENDONUCLEASE 1* (*FEN1*), *ORIGIN RECOGNITION COMPLEX 2* (*ORC2*), *REPLICATION FACTOR C3* (*RFC3*), *REPLICATION FACTOR C1* (*RFC*) and *DNA POLIMERASE ALPHA SUBUNIT A* (*POLA*), encoding putatively interacting proteins. **d**, **e**, Cluster “negative regulation of gene expression” (GO: 0010629 FDR pval 4.90E-02), including genes such as *RNA-DIRECTED DNA METHYLATION 3* (*RDM3*), R*NA-DIRECTED DNA METHYLATION 1* (*RDM1*), *JUMANJI 25* (*JMJ25*) and *JMJ30*, *ARGONAUTE 1* (*AGO1*) and *AGO10*, and *DNA-DIRECTED RNA POLYMERASE D SUBUNIT 2B* (*NRPD2*). These results are in line with observations in Arabidopsis, maize and wild tomatoes, where imprinted genes are enriched for putative chromatin modifying functions or epigenetic regulators and for genes with known functions in seed development (Gehring et al., 2011; Waters et al., 2013; Pignatta et al., 2014; Roth et al., 2018). **e** Cluster “seed development” (GO: 0048316, FDR pval 3.80E-02), which included genes such as *OCTOPUS* (*OPS*), *TOPLESS* (*TPL*), *BRASSINAZOLE-RESISTANT 1* (*BZR1*), *DEMETER* (*DME*), *LATE EMBRYOGENESIS ABUNDANT* (*LEA6*), *MATERNAL EFFECT EMBRYO ARREST 13*, (*MEE13*), among other important seed development regulators. The enriched GO terms and additional details can be found in **Table S1**.


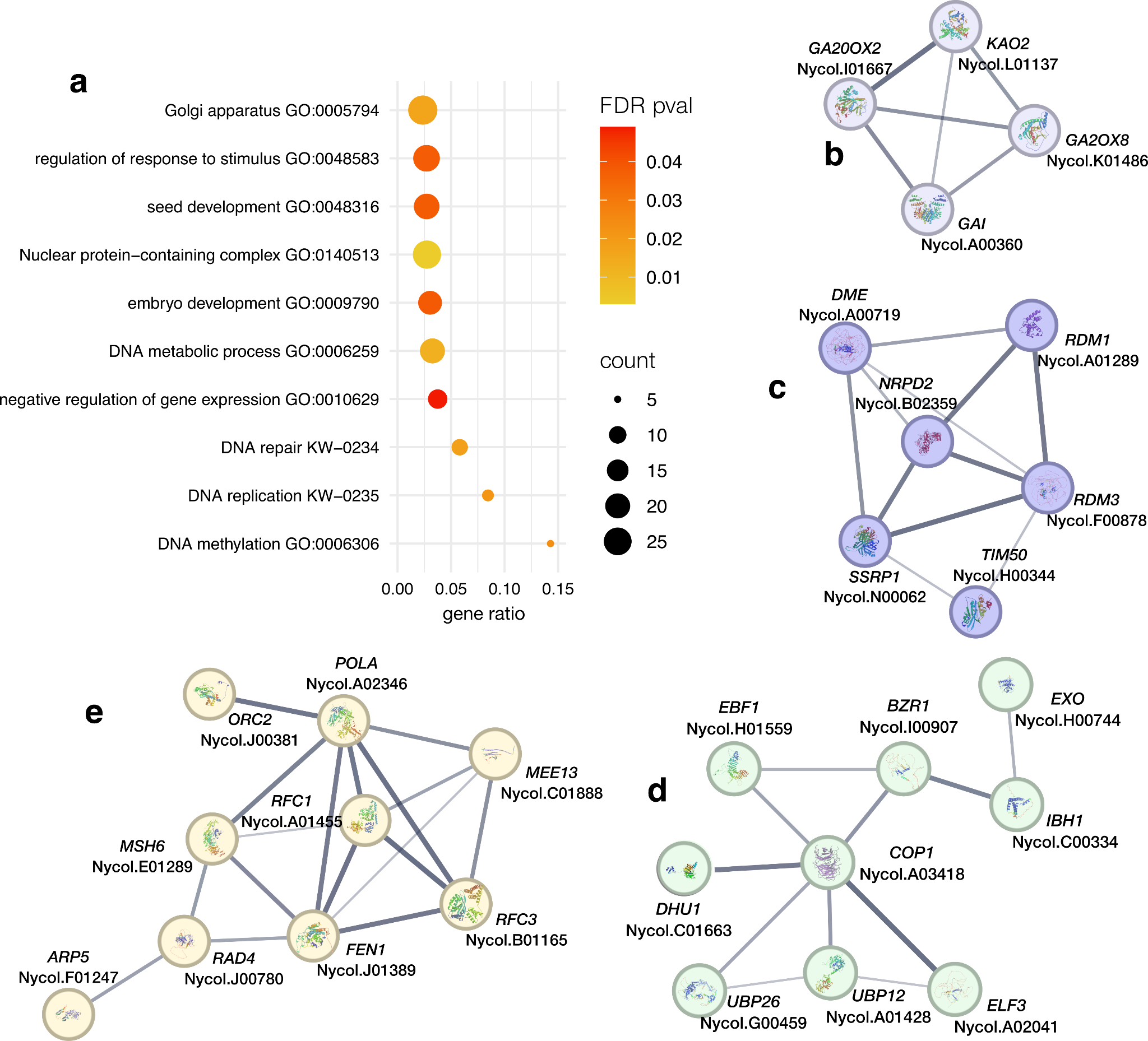


**Fig. S3 Delimitation of tissue-specific genes based on the intersection of differential expression in pairwise tissue comparisons**. The heatmaps and Venn diagrams show the expression patterns and intersected groups of differentially expressed genes (DEGs) for the following tissues: **a**, 123 embryo, **b**, 150 perisperm, **c**, 665 endosperm-specific genes.


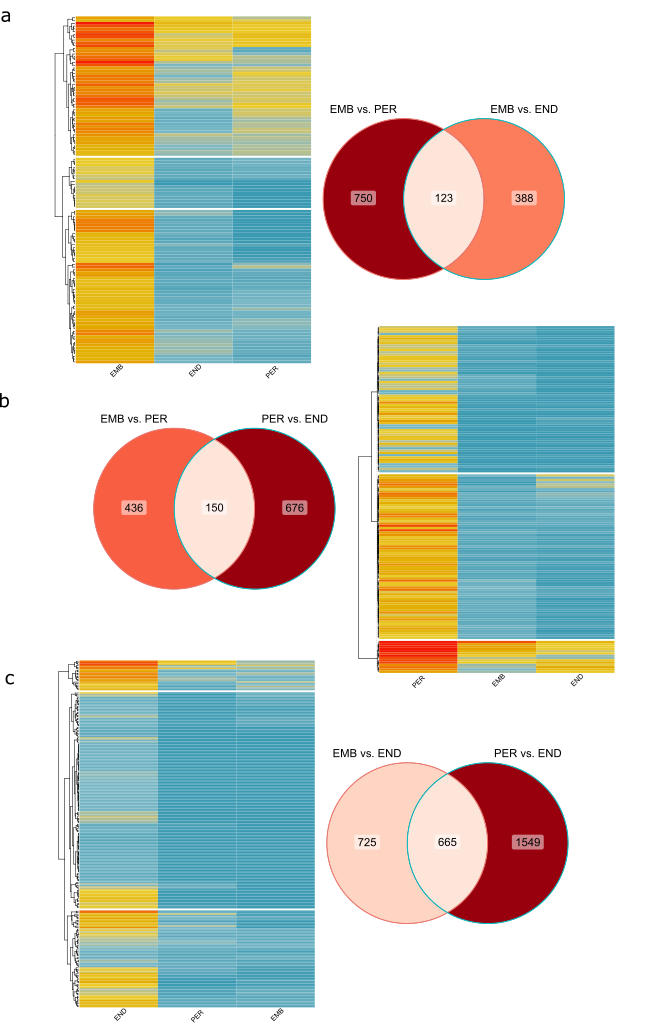


**Fig. S4 Manual annotation of cell clusters based on LCM expression patterns**. Dot plots of gene expression of LCM-derived preferentially expressed genes that overlap with the rank genes of the scRNAseq tissue clusters. From top to bottom: embryo, perisperm and endosperm. Darker colors symbolize the mean expression in the cluster and the size of the dot represents the proportion of cells in the cluster in which the gene is expressed. Full data of rank genes per cluster in **Table S4**.


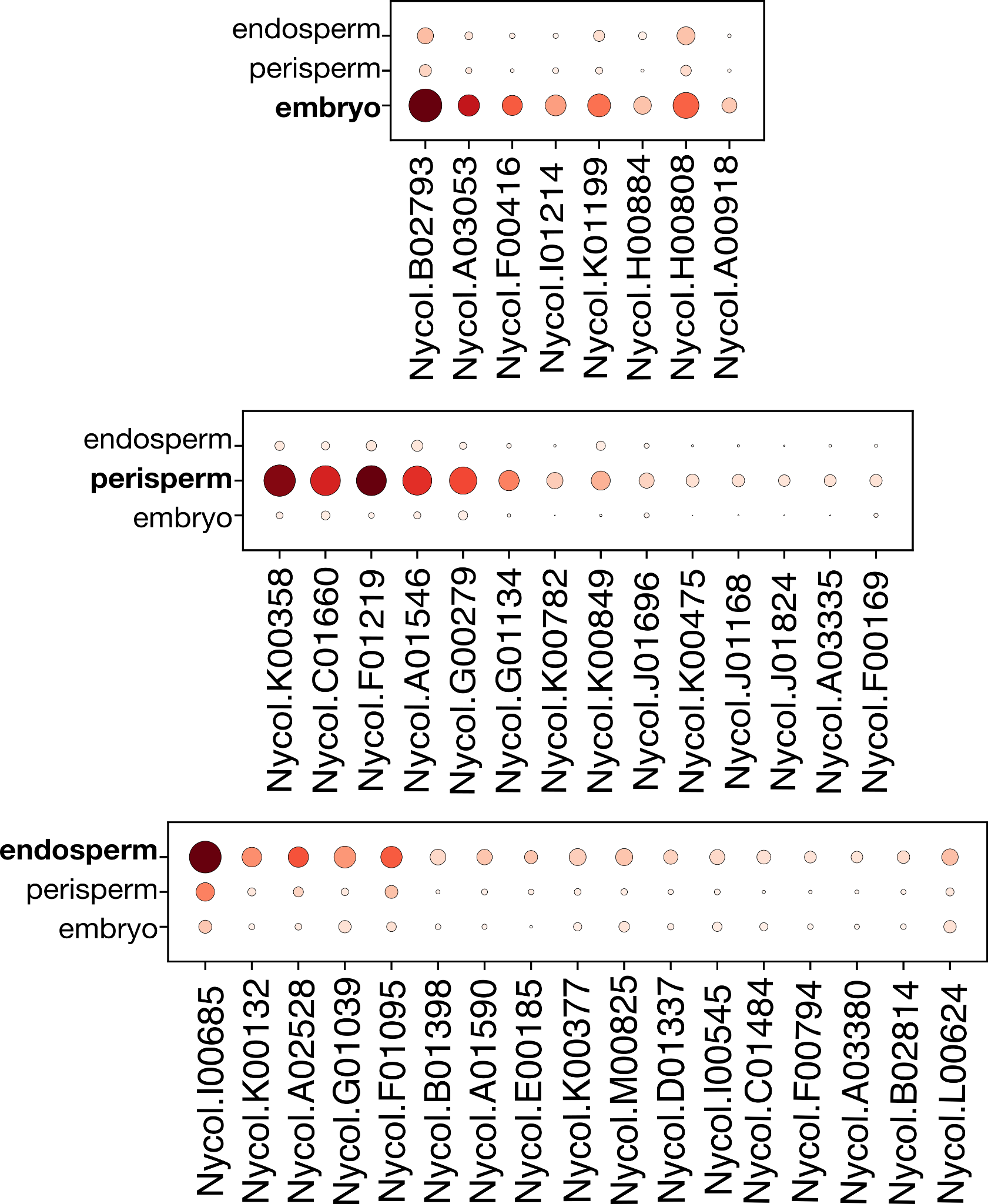


**Fig. S5 Single-cell trajectory and pseudotime analysis of gene expression in the seed compartments of *N. caerulea*, focusing on auxin-related genes. a,** Pseudotime plot where the red-to-blue color gradient reflects the continuous transition of gene expression states, with red indicating the initial expression profiles and blue marking more advanced changes along the trajectories. **b-c**, Additional trajectories inferred by Palantir, illustrating the transition of gene expression from the perisperm to the endosperm. Dots are colored according to entropy estimates. Below, heatmaps depict the dynamic expression of auxin-related genes across the trajectories.


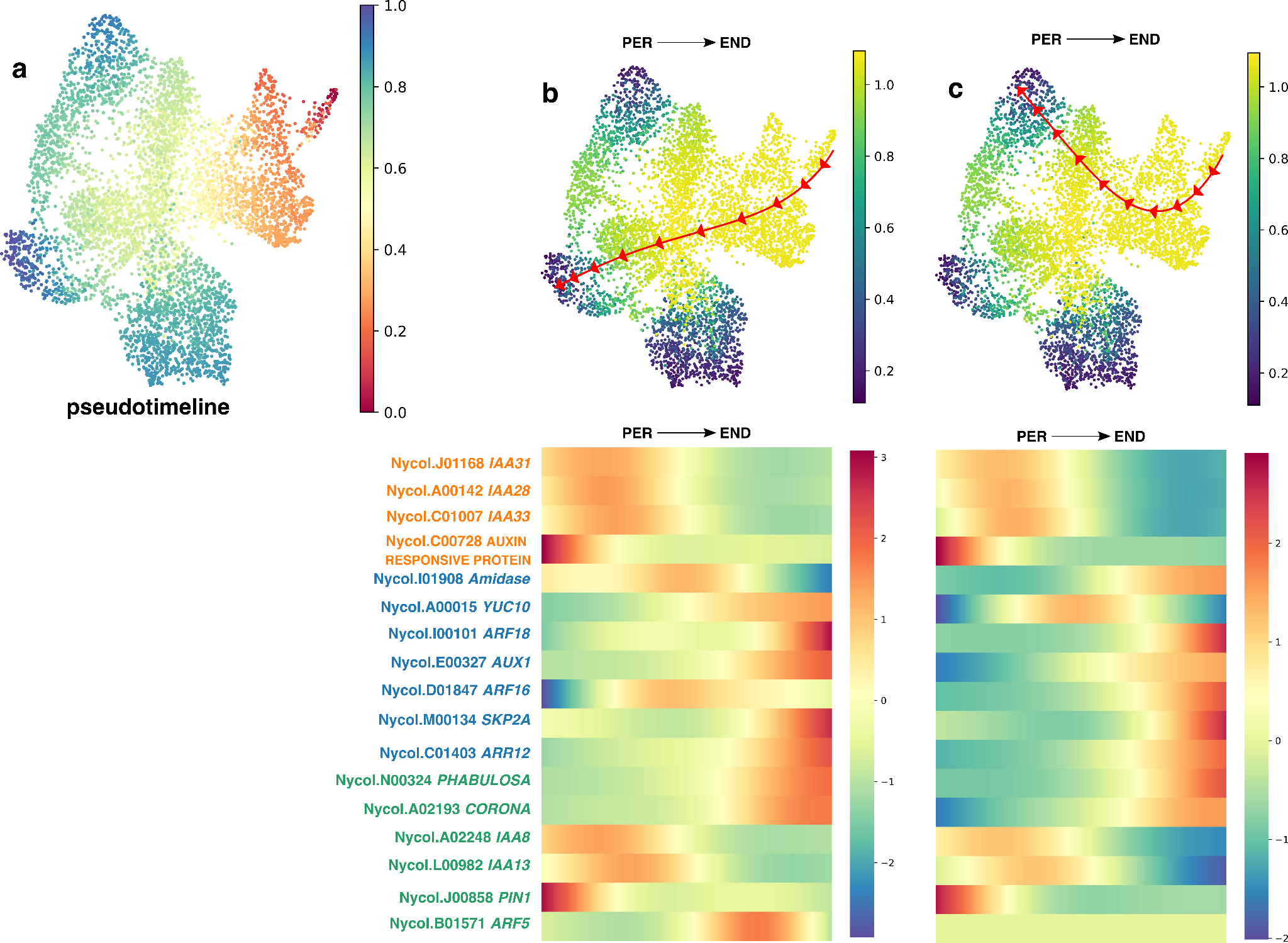


**Fig. S6 Expression of auxin related genes at the single cell resolution in the *N. caerulea* seed**. UMAP visualization of all nuclei sequenced color coded by levels of expression, from low -cold colors- to high -warm colors–. Sets of genes are grouped according to the tissue they are preferentially expressed and this is reflected on the distribution of the expression across the UMAP.

**
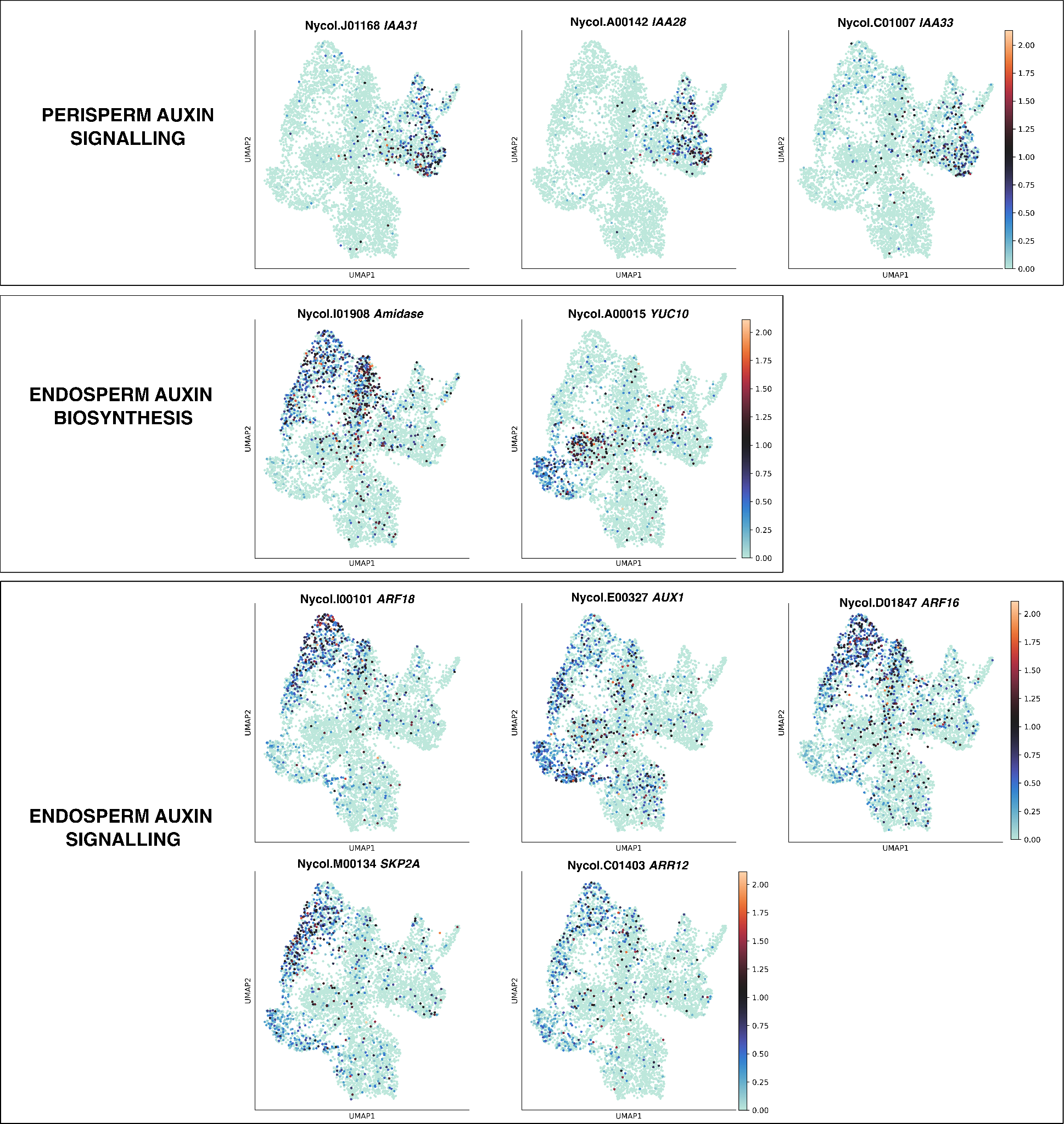
**

**
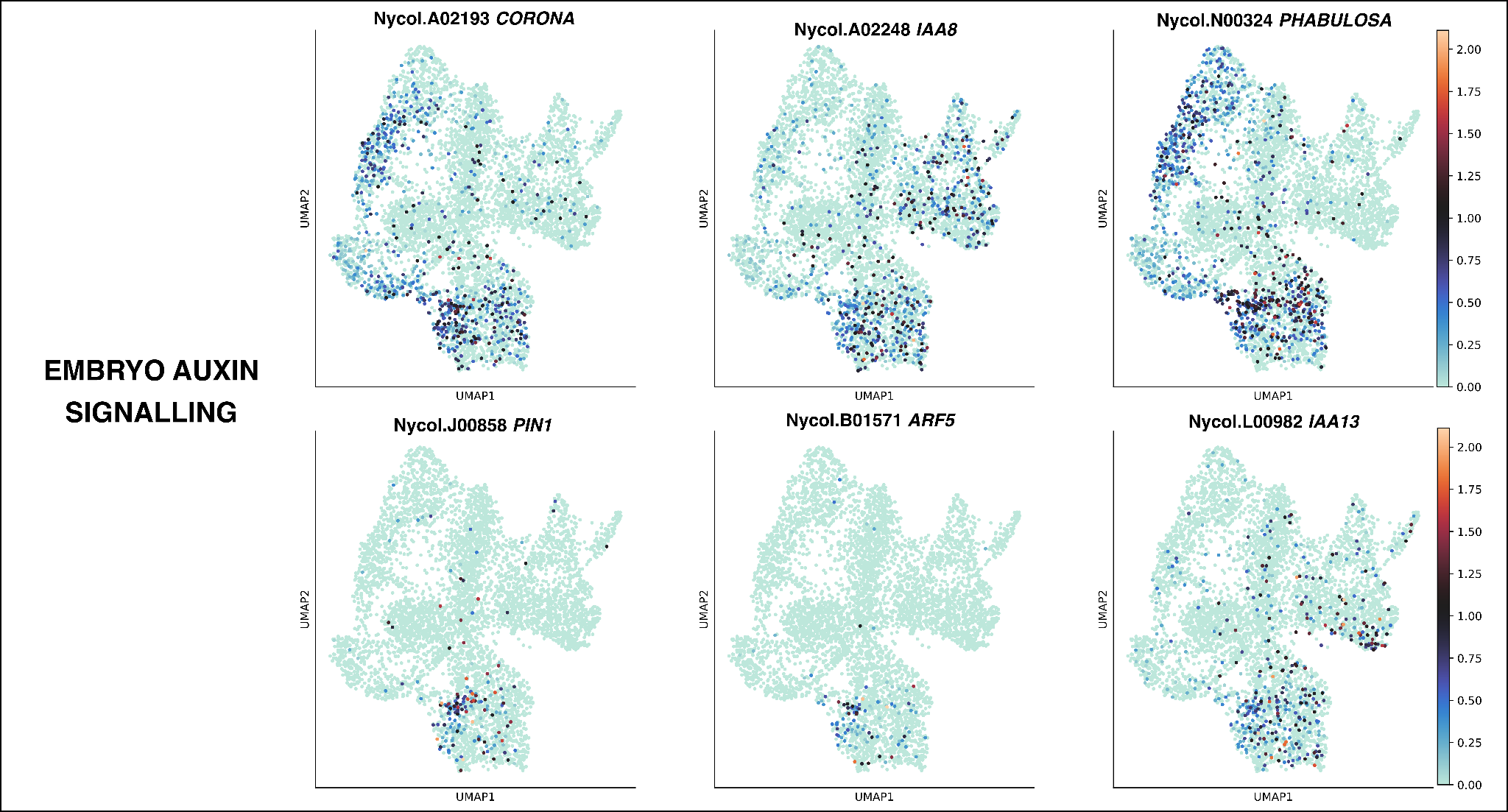
**

**Fig. S7 Auxin accumulates after fertilization in Nymphaeales seeds.** **a-b,** Comparative profiling of IAA in somatic tissues and seeds of species of *Nymphaea* (**a**) section Brachyceras, and of *Victoria cruziana* (**b**). IAA levels increase as seed development progresses and its levels in early seeds are similar to those of somatic tissues. Each data point corresponds to a biological replicate (in case of seeds, each datapoint is one seed). Letters indicate Wilcoxon rank sum test, P < 0.01.


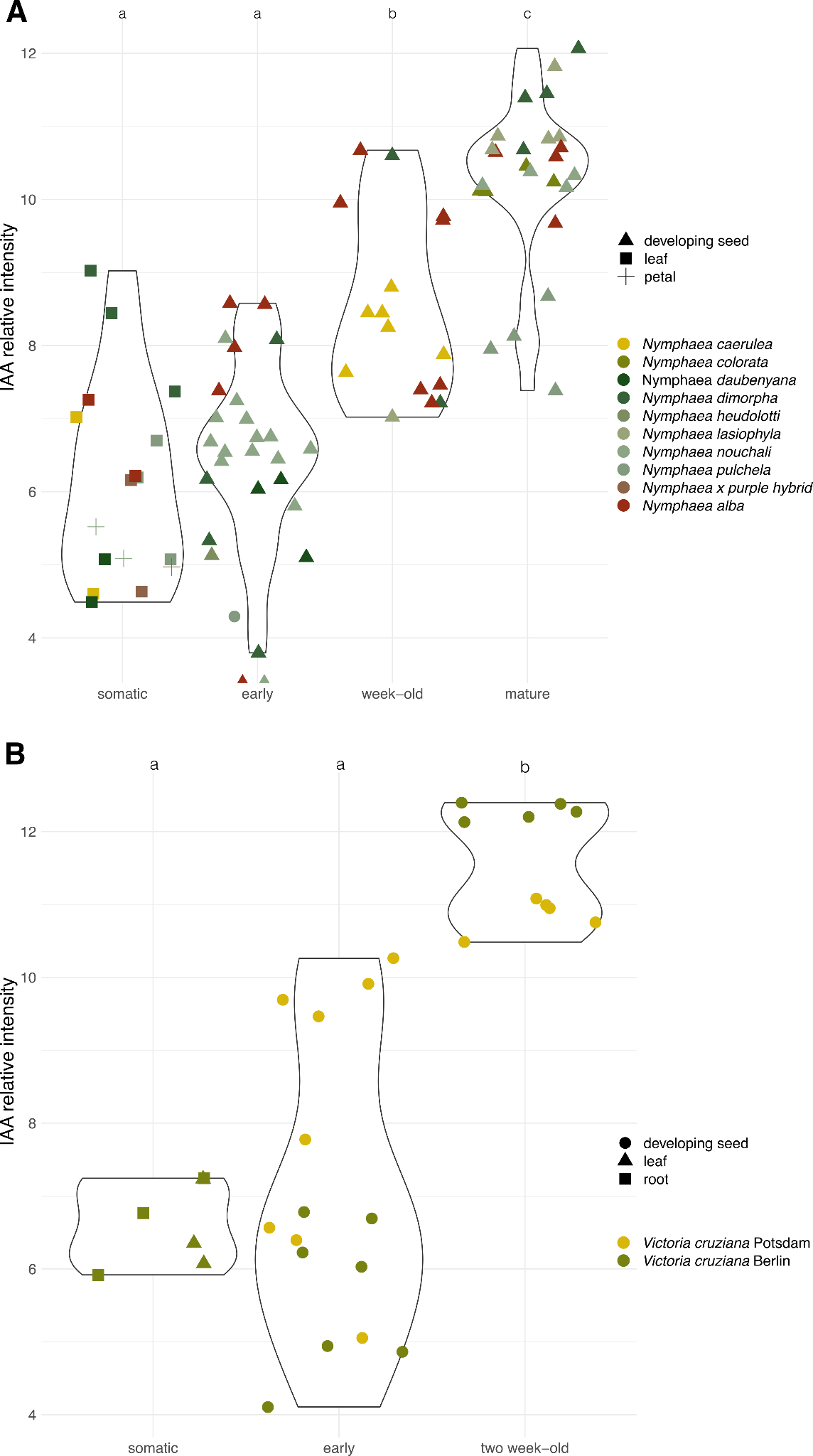


**Fig. S8 Auxin experiments in an array of Nymphaeales including species of *Victoria* spp. and in the austrobaileyale *S. chinensis***. Note how exogenous auxin application triggers seed development without fertilization independently of taxa. Mock controls are in black, auxin treatments in cool colors (green-blue) and fertilized seeds in warm colors (yellow-red). Each bar represents one fruit and each dot one seed. Fertilized seeds were not assessed for *N. purple hybrid*, *Victoria* and *Schisandra* due to the lack of compatible fathers. Asterisks and letters indicate Wilcoxon rank sum test, P < 0.01. The horizontal bar indicates the median, and the error bars indicate standard deviation.


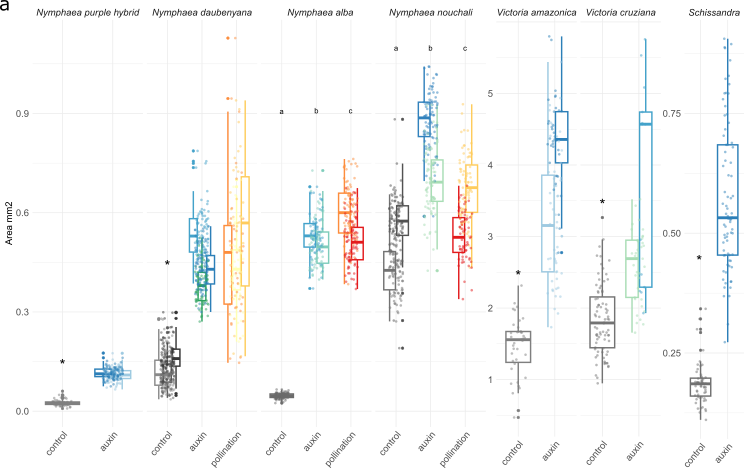


**Fig. S9 Shared gene expression in auxin-treated and pollinated tissues**. **A**, Volcano plots showing differentially expressed genes in auxin-treated ovules versus untreated controls at the day of anthesis (left) and in pollinated seeds versus the same controls (right). Points represent genes plotted by log₂ fold change (x) and –log₁₀ adjusted *p*-value (y). Point size scales with significance; grey points are non-significant. **B**, Venn diagram depicting the overlap between the auxin-treated and pollinated sets of differentially expressed genes. A total of 129 genes are shared between the two sets (set 1: n = 1,097; set 2: n = 716; universe = 20,327 expressed genes), corresponding to an odds ratio of 4.23 (Fisher’s exact test p = 7.46 × 10⁻^35^). **C,** Selected protein clusters of overlapping genes. Clusters are color-coded: blue for auxin-related genes, red for transporters, and green for transcription factors.

**
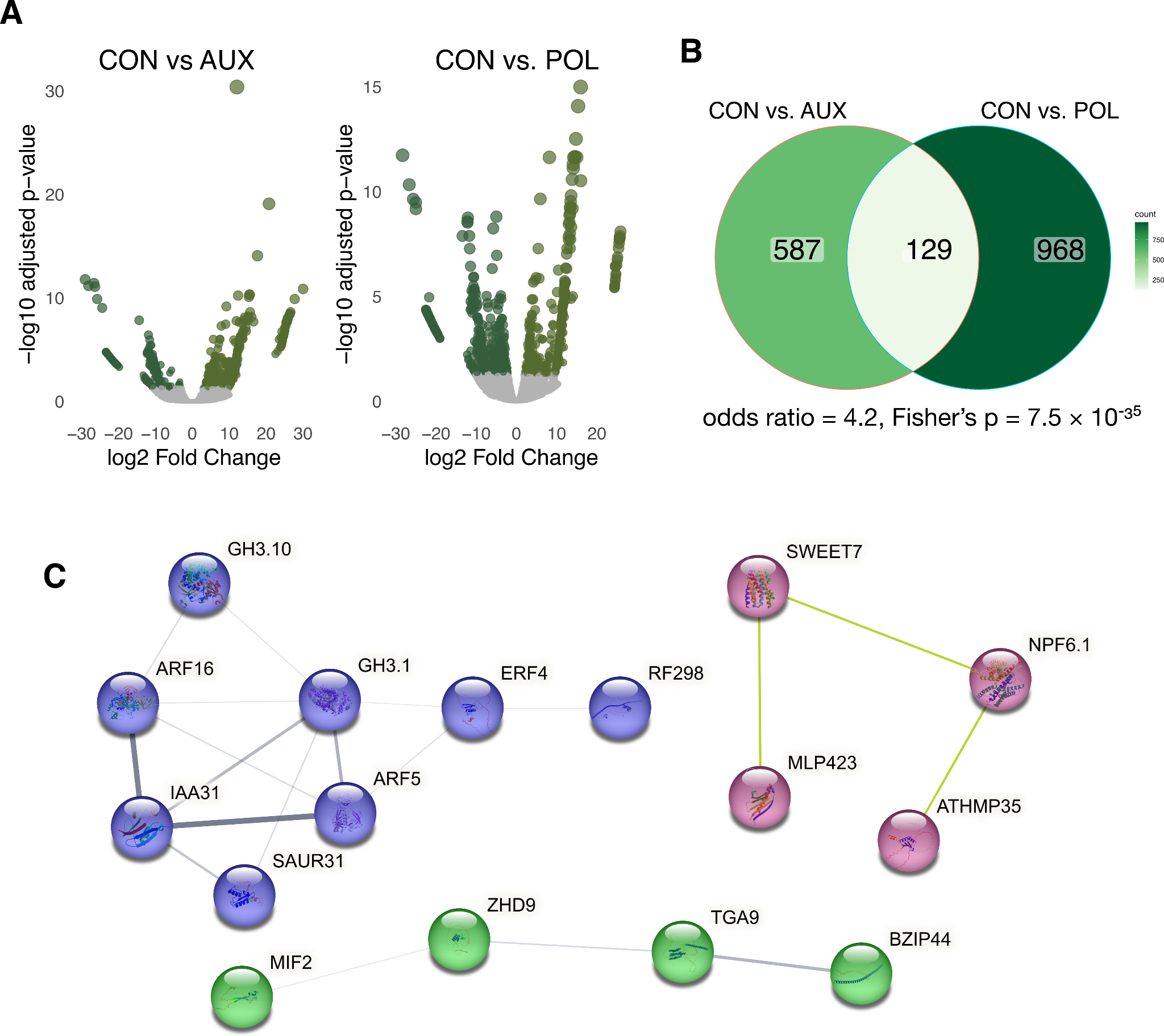
**

**Fig. S10 Principal Component Analysis of the LCM seed RNAseq datasets. “**Perisperm”, “embryo” and “endosperm” samples refer to laser capture microdissection samples of selfed seeds. “car” samples correspond to endosperms from reciprocal crosses for imprinting inference. All sample IDs match those in **Table S8**.


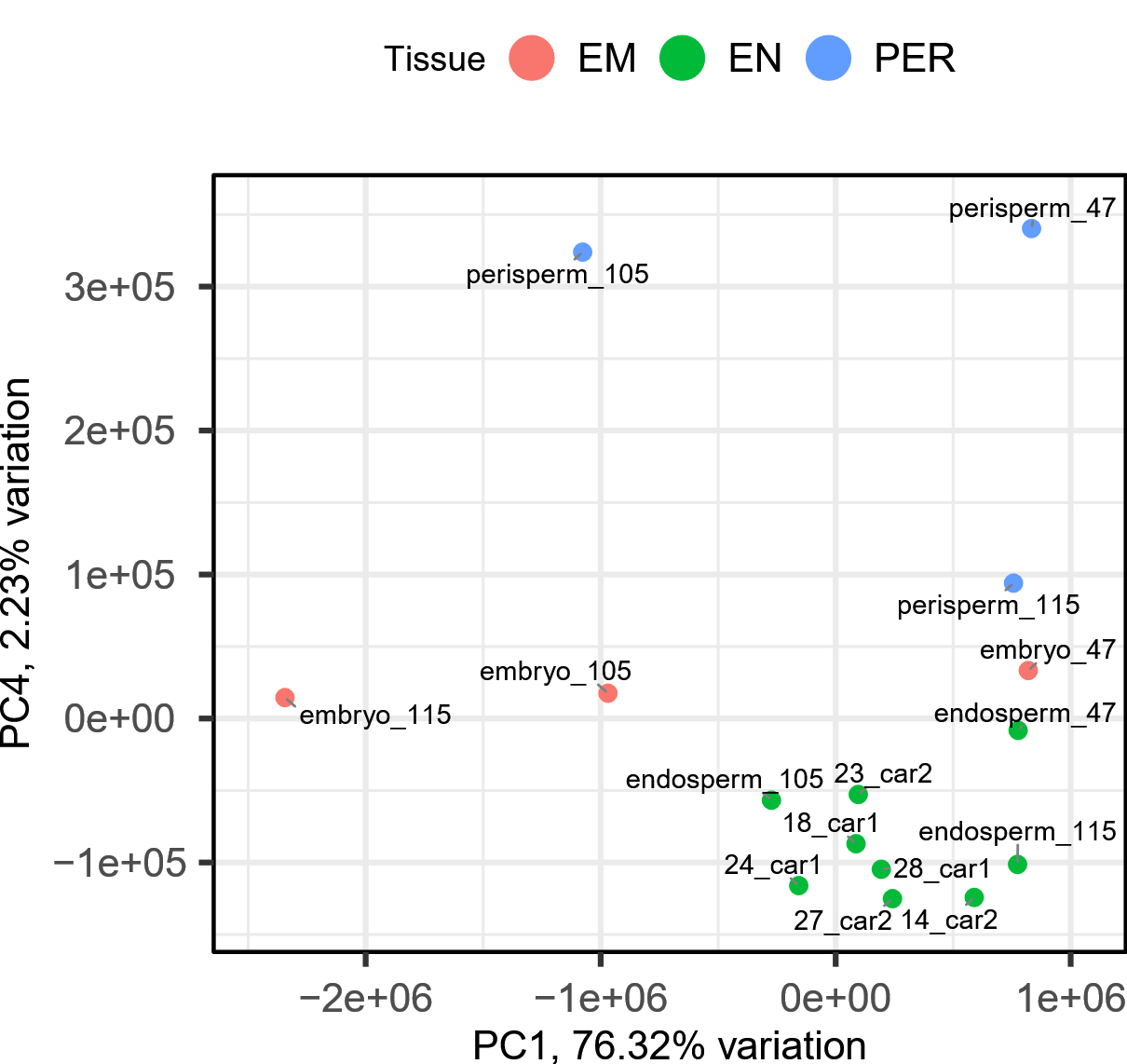


**Table S7** **Auxin biosynthesis genes have specialized in the nourishing tissues of spermatophytes.** Differential gene expression summaries of *TAR* and *YUC* ortholog genes that are enriched in the endosperms of angiosperms and in the megagametophytes of *P. pinaster* in comparison to their leaves. Data and analyses as previously published (Florez‐Rueda et al., 2024).


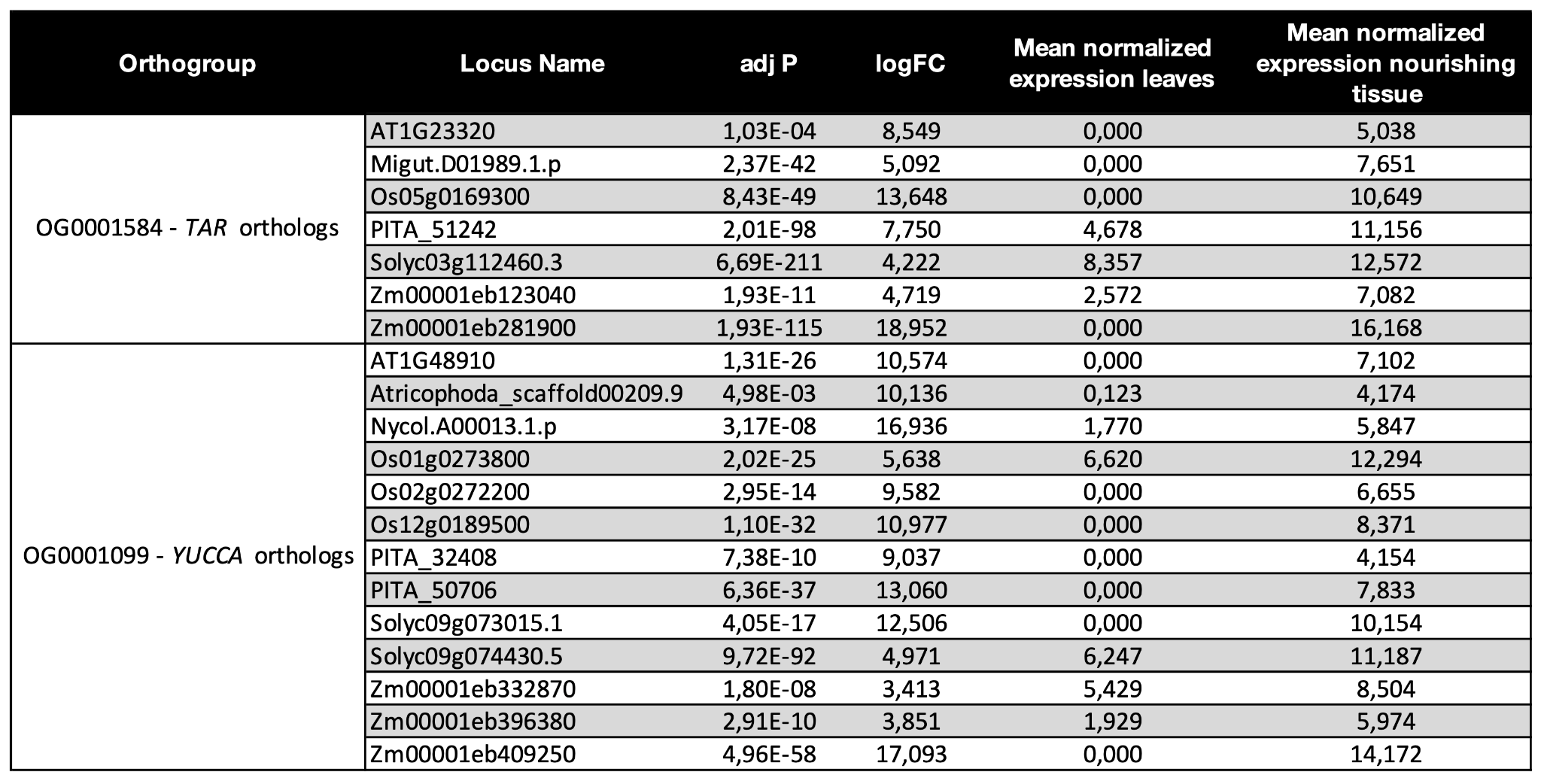


**References for the supplement**

[**Florez‐Rueda, A. M., Miguel, C. M., and Figueiredo, D. D.** (2024). Comparative transcriptomics of seed nourishing tissues: uncovering conserved and divergent pathways in seed plants. *The Plant Journal* **119**:1134–1157.](https://www.zotero.org/google-docs/?eDOouq)

[**Gehring, M., Missirian, V., and Henikoff, S.** (2011). Genomic analysis of parent-of-origin allelic expression in *Arabidopsis thaliana* seeds. *PLoS ONE* **6**:e23687.](https://www.zotero.org/google-docs/?eDOouq)

[**Pignatta, D., Erdmann, R. M., Scheer, E., Picard, C. L., Bell, G. W., and Gehring, M.** (2014). Natural epigenetic polymorphisms lead to intraspecific variation in Arabidopsis gene imprinting. *eLife* **3**:e03198.](https://www.zotero.org/google-docs/?eDOouq)

[**Roth, M., Florez-Rueda, A. M., Paris, M., and Städler, T.** (2018). Wild tomato endosperm transcriptomes reveal common roles of genomic imprinting in both nuclear and cellular endosperm. *Plant Journal* **95**:1084–1101.](https://www.zotero.org/google-docs/?eDOouq)

[**Waters, A. J., Bilinski, P., Eichten, S. R., Vaughn, M. W., Ross-Ibarra, J., Gehring, M., and Springer, N. M.** (2013). Comprehensive analysis of imprinted genes in maize reveals allelic variation for imprinting and limited conservation with other species. *Proc Natl Acad Sci U S A* **110**:19639–19644.](https://www.zotero.org/google-docs/?eDOouq)
